# Editorial: Environmental Genomics and Epigenomics: Response, Development and Disease

**DOI:** 10.3389/fgene.2021.694288

**Published:** 2021-05-20

**Authors:** Yanqiang Li, Hyang-Min Byun, Timothy M. Barrow, Qiang Zhang

**Affiliations:** ^1^Basic and Translational Research Division, Department of Cardiology, Boston Children's Hospital, Boston, MA, United States; ^2^Department of Pediatrics, Harvard Medical School, Boston, MA, United States; ^3^Population Health Sciences Institute, Newcastle University, Newcastle upon Tyne, United Kingdom; ^4^Faculty of Health Sciences and Wellbeing, University of Sunderland, Sunderland, United Kingdom; ^5^Department of Occupational and Environmental Health, School of Public Health, Tianjin Medical University, Tianjin, China; ^6^Center for International Collaborative Research on Environment, Nutrition and Public Health, School of Public Health, Tianjin Medical University, Tianjin, China

**Keywords:** environmental exposures, DNA methylation, histone modification, epigenetics, disease

With the rapid process of industrialization and the growth of the human population to unprecedented levels, individuals are increasingly exposed to multiple pollutants known to have an adverse impact upon human health, such as heavy metals, endocrine disruptors, tobacco smoke, polychlorinated biphenyls, diesel exhaust particles, pesticides, and other indoor and outdoor pollutants. Multiple non-communicable diseases (NCDs), including stroke, heart disease, cancers and chronic respiratory disease, now amount to nearly two-thirds of the total deaths caused by unhealthy environments. Considering this global burden, it is vital to understand the mechanisms underpinning the associations between environmental exposures and NCDs. Many studies have demonstrated that the epigenome can be altered by the environmental exposures, but the functional role of these changes in the processes of diseases have not been fully elucidated. This Research Topic has collated high-quality studies in the form of five reviews and four original research papers covering the genetic and epigenetic response to a range of environmental exposures.

Phthalates, esters of phthalic acid, are widely used as a plasticizer for a range of consumer products, but their presence in the environment has become a public health concern as they have potential effects on reproduction, development and obesity. Dutta et al. present a comprehensive review of the epigenetic changes associated with phthalate exposures. They summarized the metabolism of phthalates, and the effect of phthalates on different tissues including embryonic stem cells, peripheral blood, placenta, cord blood, and germ cells. They also described the impact of phthalate exposure across the lifespan, and specifically reviewed the epigenetic effects associated with childhood asthma, lipid metabolism disorders, obesity, men's reproductive health, allergies, and cancer. In addition, the authors summarized differential expression of microRNAs associated with phthalate exposure in gestational diabetes, female fertility, and the placenta.

Bisphenol A (BPA) is used in the production of a range of plastics, but it has a well-established impact as an endocrine disruptor. Qin et al. reviewed the recent progress in the study of epigenetic changes following BPA exposure. DNA methylation changes have now been revealed in multiple genes that are involved in brain development, cancer progression and other key cellular signaling pathways. Histone modifications also change significantly at some loci upon BPA exposure. Lastly, Qin et al. reviewed the recent studies of the toxic-epigenomics approach to study the epigenetic effect of BPA exposure at the genome-wide level.

Nano silicon dioxide (Nano-SiO_2_) has systematic toxicity, which is due to the induction of oxidative stress and inflammation in multiple organs. However, the carcinogenicity of nano-SiO_2_ has not yet been established. Lou et al. investigated carcinogenicity of nano-SiO_2_ exposure *in vitro*, finding that exposure can induce malignant transformation and global DNA hypomethylation of human bronchial epithelial cell lines. In particular, demethylation of CpG islands within the NRF2 promoter region results in increased gene expression, and this gene may have a functional role in nano-SiO_2_-associated carcinogenesis.

Wang et al. investigated the tissue- and sex-specific DNA methylation changes resulting from lead exposure in mice by applying ERRBS to the liver and blood of male and female mice. The authors identified hundreds of differentially methylated regions (DMRs) following lead treatment, with the majority of these proven to be tissue- and sex-specific. They also identified differential methylation of several mouse imprinted genes common to both tissues and genders. Together, these findings reveal the diversity in epigenetic changes resulting from lead exposure and could form the basis of future studies in humans.

Pulczinski et al. utilized a mouse model of allergic lung disease to examine the effects of pre- and perinatal house dust mite (HDM) allergen exposure across three generations. Their data suggests that maternal HDM exposure (F0) may act synergistically with adult HDM exposure, leading to enhanced airway hyper-responsiveness (AHR) and lung inflammation when compared to mice exposed solely in adulthood. These findings indicate that maternal allergen exposures are capable of enhancing either susceptibly to or severity of allergic airway disease. They explored the mechanistic basis of the role of epigenetics in the multigenerational inheritance by utilizing genome-wide MeDIP (Methylated DNA Immunopreciptation)-seq and hMeDIP-seq analyses to identify genes differentially methylated (DMG) and hydroxy-methylated (DHG), which serves to help elucidate the mechanisms governing multigenerational inheritance of asthma risk.

Many metals can induce Alzheimer's disease (AD), which is the most prevalent movement disorder and the second most common neurodegenerative disease. Cai et al. reviewed the changes in DNA methylation and histone modifications involved in AD and the contribution of the environment to its development, especially through epigenetic mechanisms. Specifically, they described the epigenetic impact of the plumbum, arsenic, and aluminum to AD. This review summarizes the relationships between Alzheimer's disease and different metals, revealing them to be an important factor in AD development and suggesting a direction for further study to develop potential epigenetic therapeutics. In parallel, Wei et al. provide a mini review summarizing the metals regulating epigenetics in Parkinson's disease. Specifically, they described the impact of lead (Pb), mercury (Hg), copper (Cu), manganese (Mn), aluminum (Al), iron (Fe), and zinc (Zn) on the epigenome, and particularly changes in DNA methylation, suggesting an important role of metal exposures in the risk of developing PD.

Most PD cases cannot be explained by genetic variation alone, indicating that epigenetic changes may have contributed to PD pathogenesis. CTCF is a chromatin architecture protein and its binding to DNA is regulated by DNA methylation and histone modifications. Freeman and Wang investigated the changes in DNA methylation and H3K27 acetylation within CTCF binding sites around several PD-associated genes whose expression was altered by exposure to rotenone, a PD toxicant. They observed a global decrease of 5-methylcytosine and increase of H3K27ac level under rotenone treatment. Local abundance of H3K27 acetylation and CTCF binding was also changed at several CTCF-binding sites in PD-associated genes. Their study has implications for the understanding of gene-environment interactions and epigenetic regulation in PD.

To understand the tool to investigate neurodevelopmental disorders, Xie et al. summarized the impact of developmental exposure on neurodevelopment, highlighting the importance of using *in vitro* neural cell system to study neurotoxicity. The authors discuss recent progress using cell culture to recapitulate brain complexity: composition, structural, and functional complexity are discussed, respectively. Finally, how to mimic developmental exposure and assess toxicity using cell culture models are discussed in the context of elucidating their role in neurodevelopmental disorders.

To summarize, our special topic covers endocrine disruptors, neuro toxins, inorganic metals in investigating DNA methylation, histone modifications and miRNAs, linking embryonic development, cancer progress, neurodegenerative diseases, using human, animal, and *in vitro* studies. These research studies complemented by review articles that summarize our current understanding of environmental epigenetics and the role of such epigenetic alterations in the development of non-communicable diseases. We hope that our Research Topic will serve to educate, stimulate debate, and guide future research.

## Author Contributions

YL and QZ prepared the manuscript with extensive help from H-MB and TB. All authors contributed to the article and approved the submitted version.

## Conflict of Interest

The authors declare that the research was conducted in the absence of any commercial or financial relationships that could be construed as a potential conflict of interest.

